# Using Flow Disruptions to Examine System Safety in Robotic-Assisted Surgery: Protocol for a Stepped Wedge Crossover Design

**DOI:** 10.2196/25284

**Published:** 2021-02-09

**Authors:** Myrtede C Alfred, Tara N Cohen, Kate A Cohen, Falisha F Kanji, Eunice Choi, John Del Gaizo, Lynne S Nemeth, Alexander V Alekseyenko, Daniel Shouhed, Stephen J Savage, Jennifer T Anger, Ken Catchpole

**Affiliations:** 1 Medical University of South Carolina Department of Anesthesia and Perioperative Medicine Charleston, SC United States; 2 Cedars-Sinai Medical Center Department of Surgery Los Angeles, CA United States; 3 Medical University of South Carolina Biomedical Informatics Center Charleston, SC United States; 4 Medical University of South Carolina College of Nursing Charleston, SC United States; 5 Department of Urology Medical University of South Carolina Carleston, SC United States

**Keywords:** robotic surgical procedures, patient safety, ergonomics, crossover design

## Abstract

**Background:**

The integration of high technology into health care systems is intended to provide new treatment options and improve the quality, safety, and efficiency of care. Robotic-assisted surgery is an example of high technology integration in health care, which has become ubiquitous in many surgical disciplines.

**Objective:**

This study aims to understand and measure current robotic-assisted surgery processes in a systematic, quantitative, and replicable manner to identify latent systemic threats and opportunities for improvement based on our observations and to implement and evaluate interventions. This 5-year study will follow a human factors engineering approach to improve the safety and efficiency of robotic-assisted surgery across 4 US hospitals.

**Methods:**

The study uses a stepped wedge crossover design with 3 interventions, introduced in different sequences at each of the hospitals over four 8-month phases. Robotic-assisted surgery procedures will be observed in the following specialties: urogynecology, gynecology, urology, bariatrics, general, and colorectal. We will use the data collected from observations, surveys, and interviews to inform interventions focused on teamwork, task design, and workplace design. We intend to evaluate attitudes toward each intervention, safety culture, subjective workload for each case, effectiveness of each intervention (including through direct observation of a sample of surgeries in each observational phase), operating room duration, length of stay, and patient safety incident reports. Analytic methods will include statistical data analysis, point process analysis, and thematic content analysis.

**Results:**

The study was funded in September 2018 and approved by the institutional review board of each institution in May and June of 2019 (CSMC and MDRH: Pro00056245; VCMC: STUDY 270; MUSC: Pro00088741). After refining the 3 interventions in phase 1, data collection for phase 2 (baseline data) began in November 2019 and was scheduled to continue through June 2020. However, data collection was suspended in March 2020 due to the COVID-19 pandemic. We collected a total of 65 observations across the 4 sites before the pandemic. Data collection for phase 2 was resumed in October 2020 at 2 of the 4 sites.

**Conclusions:**

This will be the largest direct observational study of surgery ever conducted with data collected on 680 robotic surgery procedures at 4 different institutions. The proposed interventions will be evaluated using individual-level (workload and attitude), process-level (perioperative duration and flow disruption), and organizational-level (safety culture and complications) measures. An implementation science framework is also used to investigate the causes of success or failure of each intervention at each site and understand the potential spread of the interventions.

**International Registered Report Identifier (IRRID):**

DERR1-10.2196/25284

## Introduction

### Background

The integration of technology into health care systems is intended to provide new treatment options and improve the quality, safety, and efficiency of care. Robotic-assisted surgery (RAS) is an example of high technology integration in health care, which has become ubiquitous in many surgical disciplines. RAS cases have tripled over the past decade [1] largely replacing both open and traditional laparoscopic surgeries for many common procedures [2]. Similar to many other types of technology in health care, RAS has changed tasks and workflow [3,4], demanding additional skills or training and introducing new complexities ranging from skill building and learning curves to workspace and organizational issues associated with operating room (OR) layout. Although RAS is associated with less postoperative pain [5], blood loss [6], and conversion to open surgery [5], safety incidents in RAS may be higher than in traditional laparoscopy [7], which has led to concerns about the speed of adoption and implementation [8]. Similar to other advanced technologies, the spread of RAS has preceded these system-level considerations, which are difficult to predict, so risks may not be immediately apparent and often go unaddressed [9-12].

RAS implementation focuses on establishing the technical skills of the surgeon operating via the robotic console [13]. However, the physical separation of the surgeon from the OR team also introduces additional communication challenges [14,15], which can lead to errors [16] and even patient harm [17,18]. RAS has particularly acute effects on equipment congestion, the movement paths of staff, and the safe positioning of data and power cables necessary for function [19]. The learning curve required to counter this multitude of systems integration challenges may continue in RAS well beyond those required in open surgery cases [2,19] and account for a steady increase in the experience recommended to achieve competency [7]. Thus, increasing task demands, combined with unique teamwork and communication challenges and existing workspace issues, may predispose to safety incidents in RAS. However, organizations are left to identify and resolve these risks without formal guidance and, in many cases, without available expertise to create formal solutions [7,20]. Human factors engineering techniques, which have been applied across many different industries to improve safety and performance [21], can be used to identify and alleviate risks in RAS. Using ethnographic approaches and systems analysis tools, human factors engineering seeks to enhance clinical performance through an understanding of the effects of teamwork, tasks, equipment, workspace, culture, and organization on human behavior and abilities.

As models of surgical processes have improved, it has become possible to reliably observe the disruptive effects of systems issues on intraoperative performance and their downstream effects on mortality and morbidity. For nearly 2 decades, direct observation of surgical work has been used to understand potential hazards in the surgical process [22,23]. Direct observation remains the best way to record variations in a process, the impact of system design on individual patterns of work, and the wider systems effects of implementing surgical technology. Unlike laboratory or simulated settings, direct observation allows us to distinguish between *work as done* (ie, what really happens) and *work as imagined* (ie, what should happen, what we think happens, or what we are told happens), illuminating the reality of how work is accomplished outside of an idealized expected or desired occurrence of events. In this paper, we discuss the design of a methodological framework and study execution applied to improve the processes of care in RAS.

### Study Objectives

This 5-year study will take a human factors engineering approach to improve the safety and efficiency of RAS across 4 US hospitals. The primary objective of this study is to generate a set of integrated, evidence-based tools for improving the safety and efficiency of robotic surgery by (1) improving teamwork and communication skills, (2) improving and standardizing technical tasks such as instrument changes and robotic docking, and (3) improving the working environment. The secondary objectives are to (1) understand the effects of organizational and work context on the spread of good practice in high-technology surgery and (2) generate a computational model of the mechanisms by which small, seemingly innocuous events escalate to create serious surgical complications. This will fundamentally improve our understanding of how innovative surgical technologies can be safely deployed and integrated within clinical work systems.

## Methods

### Study Design

This 6-phase study includes the observation and analysis of RAS cases sampled across 4 hospitals. The study will use a pseudostepped wedge crossover design with 3 individual interventions—teamwork training (TT), task design (TD), and workspace design (WD), introduced in different sequences at each of the 4 hospital sites over 4 phases (phases 3-6) of 8 months each. We elaborate on the proposed interventions below.

#### TT Interventions

TT interventions will be built based on teamwork training and nontechnical skills frameworks and will support the skills needed for teams to address RAS-specific communication challenges. The TT approach will consist of a TeamSTEPPS [24,25] driven training package (4- to 6-hour meeting for surgeons and anesthesiologists via small group teaching and successive 1-hour meetings for OR staff) complemented by on the spot coaching by human factors experts to offer reminders and encouragement.

#### TD Interventions

TD interventions will focus on specifying, ordering, and allocating tasks to specific roles to improve efficiency, visibility, and reliability [26]. A previously performed failure modes and effects analysis [27,28] will be used to prioritize tasks for redesign. The Systems Engineering Initiative for Patient Safety model [21] will be used to determine a human-centered systems model of each task, and task analysis will be used to define roles, sequences, and allocation. Finally, we will practice and refine these redesigns using in situ simulation trials.

#### WD Interventions

WD involves proposing and implementing new OR layout configurations to improve the use of space in RAS. OR layouts will be configured to ensure (1) the surgeon can see the patient and the team from the console, (2) the team can see the surgeon, (3) staff can move freely in the room, (4) robot docking can occur from multiple angles, (5) minimize cable tensions and trip hazards, and (6) optimization of OR equipment preparation and instrument storage. Key movement-oriented tasks will be used to plot ideal movement paths on existing room layouts, and new layouts will be proposed and tested to reduce unnecessary movement and disruption.

#### Stacking Interventions

Given the close interactions between technology, tasks, teamwork, and process [29-32], we hypothesize that multiple interventions will function synergistically. Teamwork benefits from visual cues, sightlines, and face-to-face communication [33,34]; TD benefits from improved teamwork to allow better coordination of complex, interdependent tasks [29,31,35]; and better efficacy of teamwork-related checklists [16], improved equipment storage, and visibility through better WD allows for improved task performance [30,36,37]. Thus, this study is designed to specifically test each of these interactions.

This design allows for sufficient implementation and sampling of the interventions, introduces individual components of an overall improvement strategy, and evaluates how each change contributes to a larger *whole* (Table 1).

**Table 1:** Study design.

Project phase	Phase 1	Phase 2	Phase 3	Phase 4	Phase 5	Phase 6	Analysis
Months	1-12	13-20	21-28	29-36	37-44	45-52	53-60
Medical University of South Carolina	Intervention refinement	Baseline	TT^a^	TT+TD^b^	TT+TD+WD^c^	TT+TD+WD^d^	Analysis
Cedars-Sinai Medical Center	Intervention refinement	Baseline	TD	TD+WD	WD+TD^d^	TD+WD+TT	Analysis
Marina del Rey Hospital	Intervention refinement	Baseline	WD	WD^d^	WD+TT	WD+TT+TD	Analysis
Ventura County Medical Center	Intervention refinement	Baseline	Baseline^d^	Baseline	TT+TD+WD	TT+TD+WD	Analysis

^a^TT: teamwork training.

^b^TD: task design.

^c^WD: workspace design.

^d^Control phases.

#### Power Analysis

Using multiple regression with 10 predictor variables (4 sites, 5 data collection phases, and 3 interventions and 1 baseline period) and assuming a normal distribution, 40 observations per site per time per intervention will provide at least 80% power to find a statistically significant effect of the intervention on surgery duration. Achieving this level of statistical power remains possible with 23 observations per phase per site, making our planned sample of 40 robust, should data collection be more challenging than anticipated.

### Study Setting

The study will be conducted at 4 hospitals in the United States, which include 2 tertiary centers with very different geography and demographics, a public *safety net* hospital, and a small private community hospital. The Medical University of South Carolina (MUSC) is an 864-bed level 1 trauma academic medical center in the southeastern United States. MUSC uses a Si da Vinci robot for general surgery procedures and Xi (X generation) for urology and gynecology procedures. Cedars-Sinai Medical Center (CSMC) is a large nonprofit tertiary care center with 958 beds and a level 1 trauma center designation in the western United States. CSMC currently has 7 da Vinci robots: 5 dual Xi consoles and 2 Xi single consoles. Marina del Rey Hospital (MDRH) is a 145-bed community hospital, acquired by the Cedars-Sinai Health System in 2018. The hospital has a small yet active robotic surgical program dating back to 2012. There is one Si robot that is used daily (up to 10 cases weekly). Ventura County Medical Center (VCMC) is a designated level 2 trauma center *safety net* hospital in the western United States and acquired its first da Vinci robot (Xi) in 2017, which is actively being used in general surgery, urology, and gynecology.

At MUSC, CSMC, and MDRH, we will sample from the following RAS procedures: urogynecology (sacrocolpopexy with and without hysterectomy), gynecology (hysterectomy for benign and malignant conditions), general and colorectal surgery (colon resection, abdominal wall hernia repair, hiatal hernia repair), bariatric (sleeve gastrectomy), and urology (simple and radical prostatectomy and nephrectomy). These cases are performed with enough volume to facilitate comparison through statistical analysis. At VCMC, an opportunity sampling approach, in which we collect any RAS procedure available, will be used because of the low RAS case volume.

### Measures

Measures will be evaluated across 3 dimensions of RAS—individual (clinicians), process (RAS case), and system (hospital) levels—and will be collected using hospital databases, observation, surveys, and interviews (Table 2).

**Table 2:** Measures and administration.

Method and measures/and variables			Phases		Administration		Dimension
**Extraction from hospital database**							
	Covariates including perioperative duration, blood loss, conversion to open returns to the OR^a^	Intervention+baseline		Data collected for all patients in each intervention phase via hospital databases (n=680)		System	
**Direct observation**							
	Patient details (age, BMI, ASA^b^)	Intervention+baseline		Collected during direct observation in the OR (or retrospectively collected from patient’s health record)		System	
	Flow disruptions	Intervention+baseline		Direct observation of number, type, and rate per observation (n~27,200)		Process	
	Surgical phase duration	Intervention+baseline		Collected during direct observation in the OR		Process	
	Oxford NOTECHS 2	Intervention+baseline		Direct observation for each phase of surgery for surgeon, OR staff, and anesthesia subteams		Process	
	Intervention adherence metric	Intervention		Direct observation once during each surgical observation (for intervention phases (3-6); n=520)		Process	
**Surveys**							
	SURG-TLX	Intervention+baseline		Completed once per observed surgery by surgeon, anesthesiologist, circulating nurse, and scrub tech (n~2270)		Individual	
	Safety attitudes questionnaire	Intervention+baseline		Administered on web via REDCap^c^ once per phase for all RAS^d^ practitioners (n~425)		System	
	Concurrent acceptability	Intervention		Administration on web via REDCap [38] twice per intervention phase (phases 3-6) for all RAS staff and clinicians (n~950)		Individual	
**Interviews**							
	Intervention implementation facilitators and barriers	Intervention		Observations and in-person interviews conducted with a diverse sample of OR staff following the implementation of all interventions (n=8-10 individuals per site)		System	

^a^OR: operating room.

^b^ASA: American Society of Anesthesiologists.

^c^REDcap: Research Electronic Data Capture.

^d^RAS: robotic-assisted surgery.

#### Covariates

Contextual covariates of known influence include patient details (age, sex, BMI, American Society of Anesthesiologists [ASA] Physical Status classification), surgery details (procedure description, procedure category, date, robot model (S, Si, Xi), hospital, OR number, approximate room size), and personnel details (number of surgical trainees, OR staff trainees [circulating nurses and surgical technicians], and anesthesia trainees). These covariates will be collected during the observations and/or retrospectively from the patient’s electronic health record. We will also record operative and in-room time, intraoperative complications, blood loss, conversion to open surgery (which requires undocking the robot and making an abdominal incision), and returns to the OR via hospital electronic records for each intervention period.

#### Flow Disruptions

Deviations from the natural progression of a task (ie, flow disruptions [FDs]) [39] were collected throughout all phases of each operation. Data collection includes a brief description of the event observed, time of occurrence, major category, and severity. FDs will be assigned a category and severity score during observation. With respect to classification, each FD will be assigned one of 8 possible categories: communication, coordination, equipment, training, external factors, environment, patient factors, and surgical task considerations (Table 3) based on an adapted taxonomy developed by Catchpole et al [40]. Minor categories may be developed for a more granular analysis of the data following data collection. FDs will also be assigned a severity score, ranging from 0 to 2: (0: potential disruption to the process, 1: disruption to the process, and 2: increased patient safety risk).

**Table 3:** Flow disruption taxonomy.

Category	Description	Examples
Communication	Any miscommunication that impacts surgery progress	Repeat information, misunderstanding, irrelevant conversation
Coordination	Any lapse in teamwork to prepare for or conduct surgery that affects surgery flow	Equipment adjustment or reposition, personnel rotation, personnel unavailable, lack of knowledge
Equipment	Any equipment issue that affects surgery progress	Robot inoperative, equipment or /instrument inoperative, suture issues, insufflation problems
Environment	Any room conditions that impact surgery progress	Outlet positioning, untangling wires and tubing, architectural design, lighting, noise
External factors	Any interruption that is not relevant to the current case	External personnel, hospital-wide alarm, personal electronic devices
Patient factors	Any patient characteristic that impedes efficient surgery	Unexpected patient reaction, patient allergy, individual differences
Surgical task considerations	Any surgeon pauses to determine the next surgical step	Surgeon decision-making, instrument changes
Training	Any instruction given to surgical team members related to the case	OR^a^ staff training, anatomy discussion, robot technical instruction

^a^OR: operating room.

#### Surgical Phase Duration

Each RAS procedure will be evaluated throughout 5 distinct surgical phases: (1) wheels in until incision, (2) incision to the surgeon on console (including the docking process), (3) surgeon on console to surgeon off console, (4) surgeon off console to patient closure, and (5) patient closure to wheels out. The duration of each phase will be recorded by the observers during data collection.

#### Oxford NOTECHS 2

The Oxford NOTECHS 2 [41] rating system will be used to evaluate the nontechnical skills of the OR team. The scale includes 4 dimensions—leadership and management, teamwork and cooperation, problem-solving and decision-making, and situation awareness—rated on an 8-point scale. Observers will record NOTECHS ratings for each team member during the case.

#### Intervention Adherence Metric

The extent to which interventions are fully used following implementation will be assessed using the intervention adherence metric [42-44], a metric developed based on the developed interventions. It will consist of a series of observational scores (Likert and check boxes) that will be deployed during each surgical observation to evaluate the use of interventions, based on observable components of each intervention. This will be deployed uniformly at baseline and all intervention phases, allowing us to understand the use of intervention during each operation.

#### SURG-TLX

Subjective workload ratings will be obtained using the SURG-TLX (Task Load Index) [42]. This visual-analog workload measure asks each surgical team member to select a score from 1 to 20 on 6 parameters: mental demands, physical demands, temporal demands, task complexity, situational stress, and distractions, which are then aggregated and rescaled to generate a workload score between 0 and 100.

#### Safety Attitudes Questionnaire

Safety culture will be assessed using the Safety Attitudes Questionnaire (SAQ) [45], which has been extensively used for nearly 2 decades. The teamwork subscale has been sensitive to teamwork interventions [46], whereas the perceptions of management subscale has identified barriers to such interventions [43]. This will be administered via REDCap (Research Electronic Data Capture, a web-based Health Insurance Portability and Accountability Act–compliant survey platform) [38] in the last 2 weeks of each data collection phase to all staff involved in robotic surgery during that trial period. Subanalysis via surgical specialty and specific operations performed will allow us to track subtle changes over time.

#### Concurrent Acceptability

To gauge team members’ responses to the interventions, we will administer the concurrent acceptability [44] measure (7 items, 5-point Likert scale) to all involved staff after the first month and at the end of the last month of each intervention phase (estimate 30-50 staff per site per phase). The measure is based on the Theoretical Framework of Acceptability model (version 2), which reflects the extent to which people deliver or receive a health systems intervention consider it to be appropriate based on anticipated or experiential cognitive and emotional responses to the intervention.

### Study Procedures

#### Observer Training

Ensuring observers are effectively trained to perceive FDs and collect data on teamwork above the *noise* of otherwise normal system function is a critical requirement for this study [23]. During the prebaseline phase, observers will receive extensive training that includes initial classroom instruction (human factors and FD classification frameworks) and practice and familiarization (eg, identification of OR team members and the components of the operating room environment) in the OR with 2 human factors researchers with extensive experience with direct observation and FD measurement in surgery. Observers will be trained to understand the basic steps for each surgery type and are familiarized with the surgical subspecialties and components of the surgical robot. Trainees will also be provided with relevant reading material on FDs, NOTECHs, and RAS and given an example of a completed data collection tool.

Familiarization observations will take place across 3 stages: (1) orientation to the OR, (2) practice observations, and (3) simultaneous observation of interrater reliability (Table 4). Weekly meetings, including the observers and principal investigators, will be initiated to combat drift and allow observers to review their observations with the team.

**Table 4:** Observation protocol.

Observation stage	Number of observations	Description
Orientation	1	Trainee and trainer observe one full RAS^a^ procedure together. This serves to orient the trainee to the OR^b^ Trainer demonstrates the following behaviors: Checking in with the charge nurse before entering OR Checking in with circulating nurse on entry to OR Where to stand in the OR and what to avoid Discuss different personnel and steps of the procedure Trainer engages in postobservation discussion Discussion of individuals in the room Answers any questions the observer had
Practice	3	Trainee and trainer observe 3 full RAS procedures together, each using the data collection tool but without discussing their observations with one another Debrief after surgery Trainer to read off their observations and times at which they observed FDs^c^. Concurrently, trainee checks observations they caught and discussion of those that they did not
Interrater reliability	5	Trainee and trainer observe 5 observations for interrater reliability If IRR^d^ (kappa>0.7) observers were considered trained and they could observe independently

^a^RAS: robotic-assisted surgery.

^b^OR: operating room.

^c^FD: flow disruption.

^d^IRR: interrater reliability.

#### In-Services

Before conducting observations, 15-minute in-services will be conducted with the staff on each unit at each study site to explain the research, introduce them to the research team, and allow them to ask questions and express their concerns. In-services will be led by a human factors expert and surgeon team member(s). Furthermore, an information sheet will be provided to staff to educate them about the purpose of the study and provide contact information for members of the study team whether they have any questions.

### Data Collection

#### Observations

A total of 4 trained human factors researchers will observe 680 RAS cases over the course of the study period. For MUSC, CSMC, and MDRH, observers will capture 40 cases during each of the 5 data collection phases (phases 2-6, each 8 months in duration). For VCMC, 40 cases will be captured in each of 2 phases: baseline (which spans across phases 2-4) and intervention (phases 5 and 6; Table 5).

Observers will collect FDs, NOTECH ratings, and all relevant case-related covariates, including patient details (age, sex, BMI, ASA classification), surgery details (procedure description, date, hospital, OR number, room size), personnel details (number of surgical trainees by type, OR staff trainees, and anesthesia trainees by type), and robot details (S, Si, or Xi model). During the intervention phases, the intervention adherence metric will also be collected during each surgical observation to evaluate the use of interventions.

Field notes will also be collected monthly by the observers at each of the 4 sites. Field notes generally consist of 2 parts: descriptive and reflective information. Descriptive information attempts to accurately document factual data (eg, date and time) and the settings, actions, behaviors, and conversations observed. Reflective information documents your thoughts, ideas, questions, and concerns as you are conducting the observation. These notes will provide additional context for the implementation of the intervention using the Consolidated Framework for Implementation Research (CFIR) [47].

Data will be collected in the OR using Microsoft Surface Pro 6 tablets. Urban Armor Gear Hand Strap & Shoulder Strap Military Drop Tested Cases are also used to provide ergonomic support and handling of tablets for observers standing or seated on stools for long period. XCOREsion 15-45 by J-Go Tech Microsoft Surface Portable Chargers were given to each observer to provide external battery life when collecting data over 2 or more consecutive cases with no opportunity to charge their tablets between cases.

**Table 5:** Data collection schedule.

Project phase	Phase 2	Phase 3	Phase 4	Phase 5	Phase 6	Total
Medical University of South Carolina	40 (5 per month)	40 (5 per month)	40 (5 per month)	40 (5 per month)	40 (5 per month)	200
Cedars-Sinai Medical Center	40 (5 per month)	40 (5 per month)	40 (5 per month)	40 (5 per month)	40 (5 per month)	200
Marina del Rey Hospital	40 (5 per month)	40 (5 per month)	40 (5 per month)	40 (5 per month)	40 (5 per month)	200
Ventura County Medical Center	13 (1-2 per month)	14 (1-2 per month)	13 (1-2 per month)	20 (2-3 per month)	20 (2-3 per month)	80
Total	133	134	133	140	140	680

#### Surveys

The SURG-TLX will be collected in person during direct observation and will be administered on a Microsoft Excel form located on the observer’s Microsoft Surface Pro tablets. The SAQ and concurrent acceptability will each be collected via a REDCap survey emailed to surgeons and OR staff.

### Postimplementation Evaluation

We will evaluate interventions as multiple case studies using in-depth interviews and observations to gain an understanding of how these process changes are adapted in each setting and what facilitates success and barriers to these changes. A diverse sample of OR managers, nurses, surgeons, assistants, and technical support personnel (n=8-10 individuals per site) will be interviewed using semistructured interview guides to elicit narratives of individual experiences surrounding RAS implementation, teamwork, surgical safety, and facilitators and /barriers to successful RAS workflow. Interviews will be guided by the CFIR [47] to examine how the interventions were implemented in each setting, considering intervention characteristics, inner and outer context, and characteristics of individuals and process involved. Our qualitative analysis will examine convergence and divergence of narratives and will present those in a case study approach. An Olympus voice recorder will be used, and audio files will be professionally transcribed. Interview transcripts and field notes will be uploaded to NVivo (QSR International, Victoria, Australia), a qualitative and mixed methods analysis software, for the analysis.

### Data Analysis

#### Statistical Analysis

We will use multivariable regression models to explore the relationship between the covariates (ie, site, specialty, BMI, and teamwork) and process measures (ie, FDs and durations), examining how these relationships are modified by interventions. The following are the specific questions we seek to answer: (1) What interventions are used (intervention adherence metric)? (2) Did OR staff like the interventions (concurrent acceptability)? (3) Did the interventions change attitudes (SAQ)? (4) Did the interventions change individual workload (TLX) and/or improve teamwork (NOTECHS)? (5) Did the interventions result in a better process (FD)? and (6) Did the interventions reduce surgical durations and/or blood loss and/or OR returns? Statistical analysis will be conducted using the R programming language (R CORE TEAM, version 3.5.2) and assessed at the significance level of α .05.

#### Point Process Analysis

Direct observation of surgical processes may be useful in modeling adverse event causation by looking at the concatenation of smaller, seemingly innocuous errors to larger, more clinically serious situations [48-50]. The primary purpose of the proposed analyses is to develop a quantitative framework that allows for the evaluation of the *snowball* hypothesis. The rationale behind this hypothesis is that accidents and injuries arise from the sequence of multiple, frequently occurring individual errors. Adverse outcomes can be seen both as the *unlucky* coincidence of multiple randomly occurring errors and/or as a causative *chain of events* where one error leads to the next, creating an error *cascade* (or *snowball*). A range of exploratory Markov chain, Poisson process, and changepoint modeling techniques will be applied with the R programming language to analyze data across more than 1000 procedures and identify error causation mechanisms as random coincidences or as a deterministic error *cascade*. This mode of analysis aims to be a profound advance, not solely in understanding and addressing surgical complications and adverse events but in the entire way in which accidents are viewed.

#### Intervention Analysis

An inductive and deductive thematic content analysis approach will be used to analyze the qualitative data [51]. In total, 2 research team members will individually code all interviews and field notes, first in a deductive pass, using a codebook to tag segments of text specific to the CFIR constructs and systems model concepts. Next, an inductive pass through the data will identify new concepts to develop themes that have not been previously identified. Codes will identify causes, explanations, relationships, patterns, and themes related to the implementation of new RAS workflows. After iterative analyses, the 2 coders will immerse and crystallize [52] the final set of themes, confirm these findings with the research team, and develop a case study of comprehensive user experiences that promote successful implementation of RAS.

## Results

### Funding and Ethics

The study was funded in September 2018 and approved by the institutional review board of each institution in May and June of 2019 (CSMC and MDRH: Pro00056245; VCMC: STUDY 270; MUSC: Pro00088741).

### Data Collection

After refining the 3 interventions in phase 1, data collection for phase 2 (baseline data) began in November 2019 and was scheduled to continue through June 2020. However, data collection was suspended in March 2020 due to the COVID-19 pandemic. We collected a total of 65 observations across the 4 sites before the pandemic. Data collection for phase 2 was resumed in October 2020 at 2 of our 4 sites.

## Discussion

### Overview

The overall goal of our research involves conducting multiple system-level interventions in RAS to validate a methodological approach to understanding and addressing latent systemic threats from new surgical technologies and measure both the effects of improvements that result as well as the utility of the interventions. Multiple interventions will be developed, tested, and planned to substantially expand our understanding of surgical safety in high-technology health care settings. This project will be the most comprehensive study to apply a human factors framework to study safety and efficiency, as it relates to technology integration in surgery. Although focused on RAS, the proposed observational, implementation, and evaluative methods of this study can be successfully applied to other health care settings integrating advanced technological systems. The study aims to address challenges and concerns using a mixed methods approach, including interviews, observations, work systems approaches, longitudinal ethnographic sampling techniques, and statistical modeling. This design is intended to capture the etiology of failure modes resulting from the mismatch between technology and existing culture. The combination of approaches will allow us to address how small, otherwise innocuous incidents can snowball into accidents and injuries in health care settings [48,49,53,54]. We also apply an implementation science framework to understand barriers to implementation, particularly of distal influences [55] or where staff may not always be supportive [7]. Understanding how and why observed effects differ among settings will allow for improved spread and sustainability. Implementing and sustaining improvements requires an ongoing involvement of stakeholders across organizational levels and boundaries [56].

Our sample includes a high volume of RAS cases performed using the da Vinci robot and conveniently sampled; this will limit the range of surgical procedures observed and will likely result in an unbalanced sample across the 4 sites. Scheduling is complex, and case cancelations and delays are an inherent deficiency in collecting observational data. The presence of the observer impacts the nature of data collected, whether as a result of implicit bias or obstructed views, and will thus affect how the data are analyzed. Although the methods described earlier are imperfect, future research teams may explore better ways to conduct these types of studies, such as through the use of video monitoring and other innovative approaches.

### Limitations

Although direct observation provides a unique opportunity to gain a true understanding of the current state of the system [16], there are challenges in conducting observational research in health care settings. These challenges include the time and effort required to train observers and organize observations [17], the costs of employing researchers to conduct observations, and the potential for the Hawthorne effect [18]. In addition, good interrater reliability among observers needs to be established and maintained throughout the course of the study, and observers need to be supported, as they may view traumatic events and feel unwelcomed in the OR [23]. Previous research has used video capture and remote video monitoring to identify teamwork, communication, and other challenges in the OR and RAS [57]. However, these methods introduce logistic and ethical challenges, including institutional review board concerns related to the identifiability of participants and data capture of adverse events. Poor fidelity and recording quality, limited viewing angles, and obstructions may also limit the usefulness of the recorded data. Moreover, the use of video recording still requires that observers conduct several videos to record observations, possibly extending the time required and expenses associated with data collection. Video capture and observations together, as was used by Randell et al [57], may represent the most comprehensive approach.

### Conclusions

This project will demonstrate the value of understanding technologies *in the wild*; the nature of partnerships between human factors experts, clinicians, administrators, and OR staff; the integration and understanding of surgical technologies; and the implications for future technological development and clinical practice. Ultimately, this study will fundamentally improve our understanding of how innovative surgical technologies can be safely deployed and integrated into complex clinical work systems. We welcome the development of similar methodologies for the evaluation and integration of various kinds of technology in health care.

## Abbreviations

ASA: American Society of Anesthesiologists
CFIR: Consolidated Framework for Implementation Research
CSMC: Cedars-Sinai Medical Center
FD: flow disruptions
MDRH: Marina del Rey Hospital
MUSC: Medical University of South Carolina
OR: operating room
RAS: robotic-assisted surgery
SAQ: Safety Attitudes Questionnaire
TD: task design
TLX: Task Load Index
TT: teamwork training
VCMC: Ventura County Medical Center
WD: workspace design
